# Long-term annual seed production data of individual European beech (*Fagus sylvatica*) trees in the Netherlands

**DOI:** 10.1038/s41597-026-07649-7

**Published:** 2026-06-23

**Authors:** Cherine C. Jantzen, Marcel E. Visser

**Affiliations:** 1 Department of Animal Ecology, Netherlands Institute of Ecology (NIOO-KNAW), Wageningen, The Netherlands; 2 Groningen Institute for Evolutionary Life Sciences (GELIFES), University of Groningen, Groningen, The Netherlands

## Abstract

Seed production of European beech (*Fagus sylvatica*) is highly variable between years and synchronised between individual trees (i.e., masting), creating years with high seed crops, commonly separated by one or more years with little or no seed production. This pulsed reproduction has selective benefits for the trees, as it creates a cycle of predator satiation and starvation, through which masting drives population dynamics of dependent species. Masting is sensitive to climate change, making long-term data on annual seed production valuable for understanding changes in the masting patterns and their effects on seed consumers. This study presents a long-term dataset of 50 years containing annual, ground-plot beechnut counts for 81 individual trees in the Netherlands. Time series length of individual trees ranges from one year to the whole study period, with 21 trees having at least 40 years of data. Beechnut counts are categorized in sound, empty and predated, complemented by gross and net weights of individual nuts, allowing for detailed analyses of masting patterns and annual variation in food abundance for seed predators.

## Background & Summary

European beech (*Fagus sylvatica*) is a forest forming tree species in Central Europe and its seeds (i.e., beechnuts) provide a valuable food resource for many forest species (e.g.^1–3^). Beeches reproduce by mast seeding (hereafter masting), meaning that their seed production is highly variable between years and strongly synchronized between individuals^4^. Consequently, beechnuts are a variable food resource, being superabundant in some years and almost absent to completely absent in other years, leading to demographic changes in seed consumer populations^1,5^. Since beechnuts are a crucial winter food resource for resident passerine birds, the dataset presented here was initially collected alongside a long-term nest box study on passerine birds^6^ to analyse the effect of winter food availability on the bird’s winter survival. It provides long-term, individual-level data on European beech seed production from the Hoge Veluwe National Park in the Netherlands ranging from 1976 to 2025. The data consist of 12296 annual ground-plot based counts and weights of individual nuts, collected under 81 individual trees.

Besides its original purpose, this dataset can contribute to our understanding of reproduction dynamics in beech and how they are affected by climate change^7^. Over the past decades, the field of masting research has grown rapidly with many comparative studies focussing on beech seed production across Europe (e.g.^8,9^) and efforts have been put into collating reproductive time-series data from a diversity of plant species into one publicly available dataset (MASTREE+^10^). Our dataset can be a valuable addition to this data collection, since the Netherlands are so far mostly not included in comparative studies, due to a lack of available long-term data. Additionally, this dataset can be used for studies around pulse ecology, since high seed years serve as a strong resource pulse, and the resulting effects on interacting species (e.g.^11,12^). Moreover, this long-term dataset can provide a baseline to capture climate change effects^7^ and can be used in forecasting studies on changes in masting under different future climate change scenarios (as done, for example, in^13,14^) or changes in consumer populations with different seed production scenarios (e.g.^15^).

Until now, the raw data, as described here, has not been used in any publications. Only an index of beechnut production derived from the raw data has been used in several studies on bird phenology and demographics, utilizing the bird data from the nest box study in the same area. The earliest study by Perdeck *et al*.^2^ showed that both juvenile and adult annual great tit (*Parus major*) survival is strongly influenced by the amount of beechnuts produced, which was confirmed in a later study using 16 years of additional data^16^. Additionally, beechnut availability has a positive effect on the probability of recruitment and the number of recruits (i.e., fledged offspring that return to breed) in great tits^16,17^. Similarly, population dynamics and fluctuations of the same species are driven by the variation in available beechnuts^18–20^ and, under simulated future scenarios of high beechnut availability, the vital rate of the birds and their population size increase^15^. In contrast, beechnut availability does not seem to have an effect on the selection for earlier egg laying in great tits^21,22^, as well as in blue tits^21^. Lastly, fluctuations in the population size of central European blue and great tit populations are synchronized by a common environmental force through large-scale synchrony in beech masting^19^.

The three-scale beech crop index (BCI) used in these studies is however only based on a subset of the original dataset and is one possible interpretation of the raw data, which in most studies is summarised at the population level. The index categorises the data based on determined thresholds (see “*Calculation of beech crop index”*), which underrepresents the amount of variation in seed production and is restricted to the number of full beechnuts. Looking at the actual counts and weights of the beechnuts can therefore give a much more fine-tuned insight into the reproductive dynamics of the beech trees.

## Methods

### Data collection

**Fig. 1:**
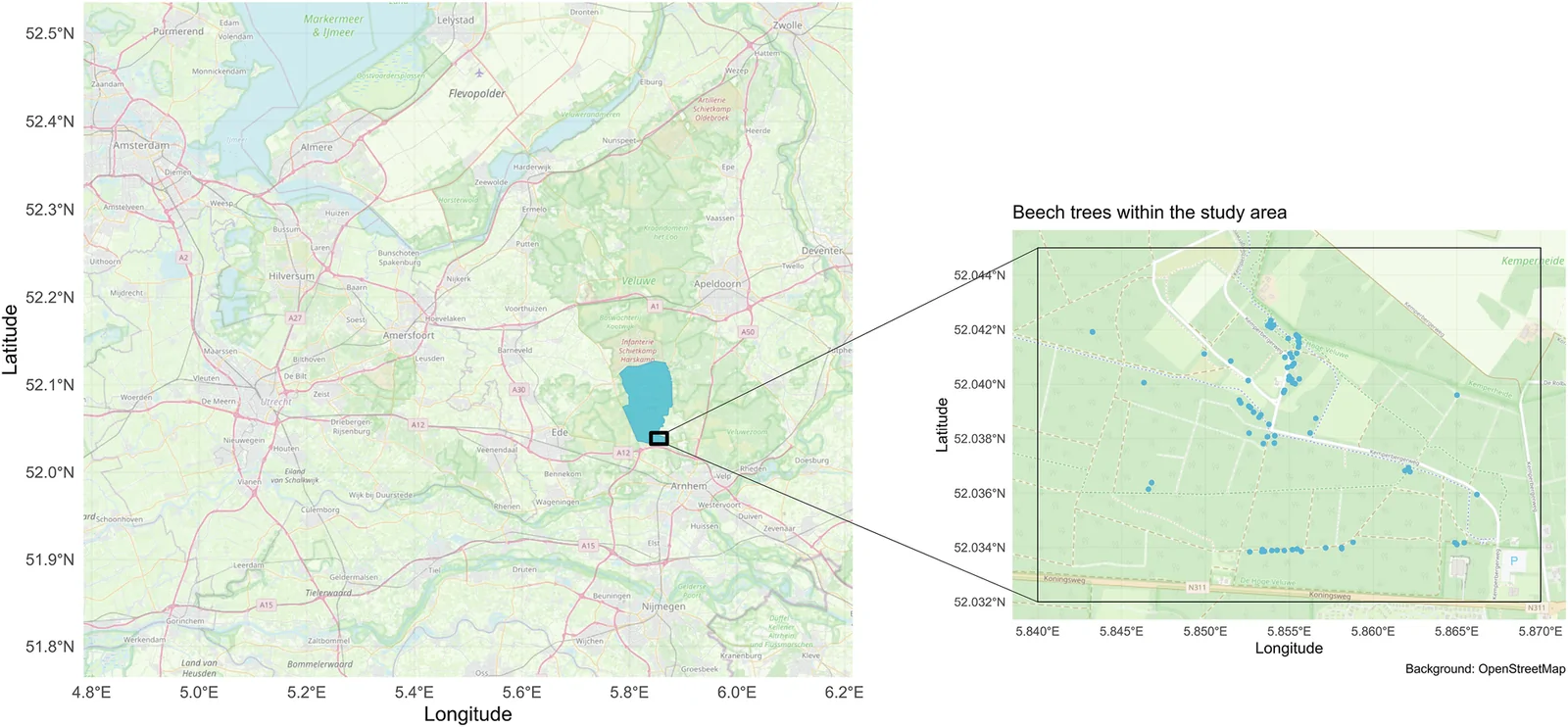
Location of the study area and the studied beech trees. The left map shows the location of the Hoge Veluwe National Park (filled in blue) on the Open Street Map of the Netherlands. The Hoge Veluwe is located north of Arnhem and part of the Natura 2000 area, as well as the Long-Term Ecosystem Research (LTER) site Veluwe. The black box indicates the location of the study area within the Hoge Veluwe. The right map is zoomed into this black box, showing the distribution of the sampled beech trees (blue dots) within the study area. Sampled trees are distributed across the whole study area where beech is present.

Beechnuts are collected annually to assess the number of nuts produced by individual trees and the net weights of the whole nuts, because the resulting total annual net weight of the kernels of the beechnuts (i.e., their fleshy part) is relevant to seed consumers. Data collection takes place in autumn, around mid-October, in the Southern part of the Hoge Veluwe National Park (52.04 °N, 5.85 °E) in the Netherlands (Fig. 1, left map), a study area of 111 ha. The study area consists of a mixed forest with oaks, birch, pine, and larch, growing on poor sandy soils.

Sampled trees are distributed across those parts of the study area that include beeches (Fig. 1, right map). Individual trees are marked and resampled every year, but deviations occur over time, as trees have died or were cut and replaced by proximate trees, or because sampling effort differed. The exact ages of the sampled trees are unknown, but documentation on forest management and the size of the trees suggest that all trees were at least 70 years old when first sampled. For the trees active in 2025, the diameter at breast height (DBH) ranged from 29 to 148 cm with a mean DBH of 65.68 cm in spring 2026 (measured at 130 cm height). Beechnuts are collected in the field with the ground-plot method, in which all nuts within defined plots are directly collected from the ground. This method cannot exclude removal of beechnuts through predation or seed dispersal before seed collection, potentially underestimating the total seed count and the level of predation. However, the ground-plot method can still be considered highly reliable in estimating seed production, especially when samples are taken shortly after the peak seed fall^23–25^. After seed collection, the beechnuts are further processed in the lab. While the general collection method stayed the same over time, the involvement of many different people in conducting the fieldwork over a period of 50 years led to slight deviations in the methodology between years, which are described in more detail in the following.

#### Field work

**Fig. 2:**
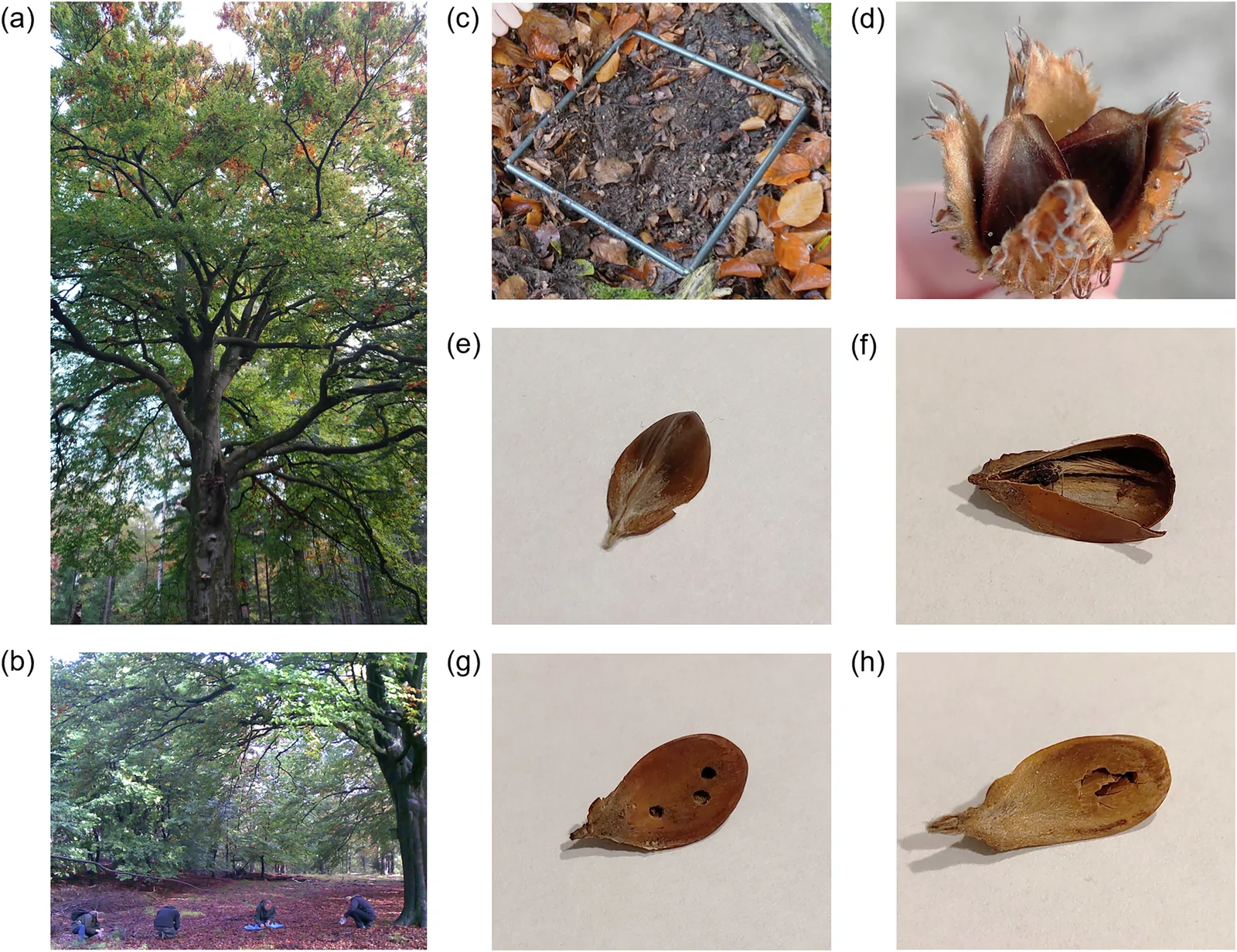
Field- and lab work of beechnut counts. (**a**) The dataset contains data of 81 beech trees (Fagus sylvatica). (**b**) Under each of them, four metal squares (25 × 25 cm; (**c**)) are placed in a straight line of a fixed direction, within which all beechnuts are collected. The first square (position 1) is placed ca. 50 cm from the trunk, the last square (position 4) underneath the tip of the largest branch, and the other two squares in equal distance in between. (**d**) One cupula (i.e., the spiky outer husk) contains two beechnuts. These cupulas are removed when collecting the nuts. (**e**–**h**) In the lab, the collected beechnuts are sorted into different categories: (**e**) full nuts that are firm and shiny on the outside, (**f**) empty nuts (here the inside of an empty nut) that are not firm but curved inwards from the outside and empty from the inside, (**g**) caterpillar nuts that show one or several round emergence holes of caterpillars, and (h) eaten nuts that show other signs of predation, like non-round holes, or signs of pecking by birds.

Beechnuts are collected by placing metal squares (hereafter plots) of 25 cm × 25 cm underneath each tree (Fig. 2a–c), collecting all full and partial nuts within the plots (if nuts are still attached to their cupula (Fig. 2d), the cupula is removed), and placing them in separate bags per plot. Plots were always placed approximately in the same direction from the tree, marked through a pin, nest box, or cross in the bark, but deviations occurred when, e.g., branches fell off. The sampling direction was initially selected to ensure little canopy overlap with neighbouring beech trees, ensuring that samples are representative for the tree they are collected from. If the same trees were sampled in several months, the plots were moved slightly left or right from the sampling direction to avoid resampling the exact same plot.

**Fig. 3:**
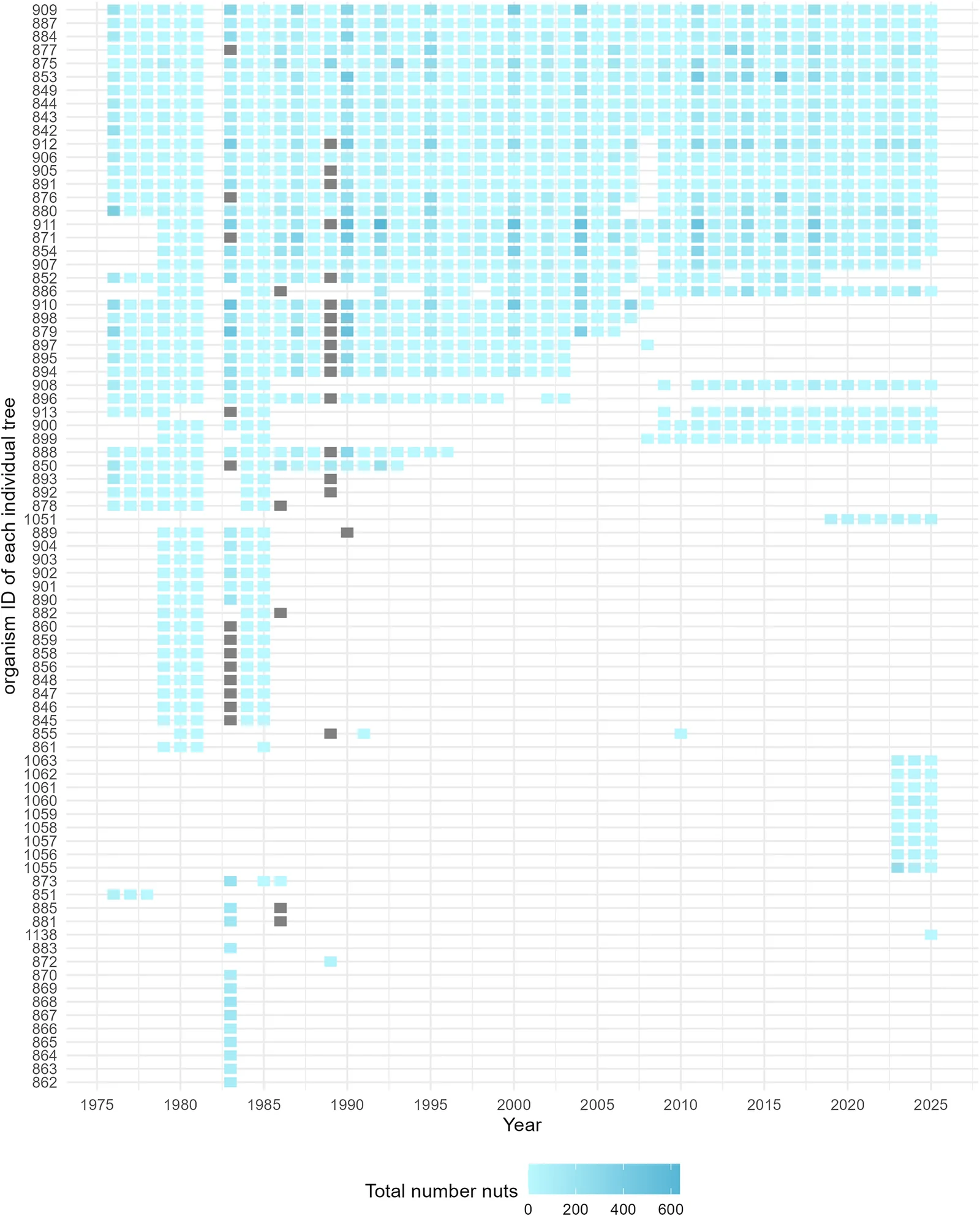
Time series length of each individual tree. While the same set of approximately 30–35 trees should be sampled each year, deviations occurred throughout the study period due to forest management practices (i.e., cutting of trees), death of trees, or differing sampling effort in some years, leading to some individuals being sampled for few years and others over the whole study period. In 1982, no data has been collected. Blue colour indicates the number of total nuts per tree per year (incl. whole, empty, caterpillar and predated nuts). As some years had several sampling dates, the total number of nuts is only calculated for the dates that are selected in the “include_dates” table. However, few trees have been sampled in some years only at dates not selected, indicated by a grey tile.

While from 1976 to 1986 three plots per tree were sampled, the number of plots increased to four from 1987 onwards. The plots were placed in a straight line with the first plot located half a meter from the trunk (position 1, see Fig. 2b), the last plot underneath the tip of the largest overhanging branch (position 4, Fig. 2b) and the remaining two plots in equal distances in between. In 2024, all plots were marked at the first sampling date (indicated in the data by SampleTypeID = 2) and these plots were re-sampled on the following sampling occasions (SampleTypeID = 3) in addition to the normal samples (i.e., SampleTypeID = 1), leading to eight measurements per tree per date (instead of four measurements). All zero values in the data represent the absence of beechnuts, and in 5 years (1981, 1988, 2001, 2005) no nuts have been found in any of the plots. Sampling effort within a season varied between years (ranging from 16 to 60 sampled trees; mean = 31.6 trees) and there is one missing year (1982), in which no data was collected. This leads to differing time series lengths per tree ranging from one year to the whole study period (mean = 19.1 years), as shown in Fig. 3.

Field work is done annually in mid-October, the standard sampling date (but sometimes more often, see below). However, in 1991 and 1998, samples were not taken in autumn but only in December and January (of the following year), respectively. While in most years, sampling was done on one day a year, there are 13 years in which sampling was spread over several sampling days. This is partly because not all trees could be sampled within one day (1977, 1995, 1979, 1980, 1986, 1992), samples were taken repeatedly within a month or across several months (1976, 1987, 1989, 1990, 2014), or both (i.e., several months have been sampled, but samples within a month were taken on two days; 1983, 2024).

#### Lab work

After the sample collection in the field, all samples are dried at room temperature for at least 24 hours. All further lab work is done per plot, i.e., tree-date-plot specific. Nuts were always sorted into whole nuts (i.e., freshly produced, firm nuts containing a developed, viable seed, Fig. 2e), empty nuts (i.e., nuts that are completely empty inside, Fig. 2f), predated nuts, which are split into nuts with caterpillars (i.e., showing small round entry holes caused by insects or containing caterpillar eggs when opened, Fig. 2g) and nuts predated upon by birds or mammals (i.e., showing non-smooth larger holes, Fig. 2h). Additionally, the categories of rotten nuts (i.e., decaying nuts, often from previous years, that contain small, dried-out black seeds) and other nuts (containing mainly pieces of nuts that cannot be put in any other category) have been used, but not in all years.

After drying and sorting, the whole nuts additionally are weighed. First, all whole nuts are weighed together to get the total gross weight and, since 1999, nuts were also weighed individually to get the weight per nut. When no individual weights were taken before 1999, the weight per nut is estimated as the mean, i.e., total weight divided by the number of nuts weighed. In high seed years, the number of nuts that have been weighed together is however sometimes lower than the total number of whole nuts that was found, so the total gross weight of all nuts is calculated by multiplying the mean weight per nut of a plot with the total number of whole nuts in the same plot. Besides the gross weight, net weights were collected by weighing the nuts after peeling of their outer shell. Since 2000, a maximum of five random nuts is weighed (gross and net weight) individually to reduce processing time. From these data the total net weight per plot can be calculated using the mean ratio between net and gross weight from these five nuts. The number of nuts that have been weighed is indicated in the data through the organismQuantity column in the occurrence table. For events describing the weighing of nuts (i.e., eventID ends on “_N” and a number; for details see data records section), the organismQuantity is either one when single nuts have been weighed, or larger than one when more than one nut was weighed together. In the latter case, there is only one weighing event (i.e., row) per plot, while there are several events per plot when several single nuts were weighed (i.e., multiple rows with identical eventIDs just differing in the running number behind the “_N”).

### Data interpretation and processing

#### Accounting for years with only three plots

For the early years (i.e., 1976–1988), in which only three plots have been sampled, the fourth position has been calculated as the mean of the three measured plots and floored to get whole counts. These entries have a remark (column “measurementRemarks”) indicating that they were calculated (i.e., “4th position extrapolated from positions 1 to 3”). This was done to make the number of plots per tree consistent, facilitating analyses.

#### Calculating net weights

To calculate the total net weight of all full nuts per year, the total gross weight (verbatimMeasurementType = “TotalGrossWeightWhole”) of all full nuts can be multiplied with the mean ratio between gross and net weight (net/gross; verbatimMeasurementType = “NetWeight”/”GrossWeight”) of the individual nuts. This calculation should be done per plot, if all three values (i.e., total gross weight, gross weight per nut, net weight per nut) are available on the plot-level. As this is however not always the case, the net-gross-weight ratio can then be calculated as follows: If either the gross or net weight of individual nuts is missing, another net/gross-ratio has to be used. If there is a ratio for the same tree (calculated from other plots at that date), the average ratio over these other plots should be used. Otherwise, the average ratio of the plots of all other trees in the same year should be used, and if that is also not available, the weighted mean of the ratio over the whole study period (being 0.61).

For some plots, the total gross weight (“TotalGrossWeightWhole”) is missing. These are however only the plots from additional sampling days outside of the normal collection in mid-October, as nuts have not been weighed for these dates. If net weights for these dates should be calculated, the average net weight per nut of the same tree on other dates in the same year can be multiplied with the number of full nuts (“NbrWhole”).

#### Including the correct dates

For the years where data has been collected on several days (see above), it is important to choose one representative date to be used for analyses that focus on annual beechnut production. Given that the standard sampling date is around mid-October, the dates closest to that should be selected, and it should be a date at which most trees have been sampled. For years, where sampling was split over several days due to time constraints, all these days have to be included. If there were several sampling events in October, the dates on which the largest number of unique trees has been sampled should be included. To facilitate selection of dates, the dataset contains an “Include_dates” table, listing all sampling dates and marking the dates to include for each year (see section “data record”). In the provided R script, that helps to convert the Darwin Core Archive (see “data record”) to a format suited for analysis, only samples of dates that should be included according to the Include_dates file are retained.

#### Calculation of beech crop index

**Fig. 4:**
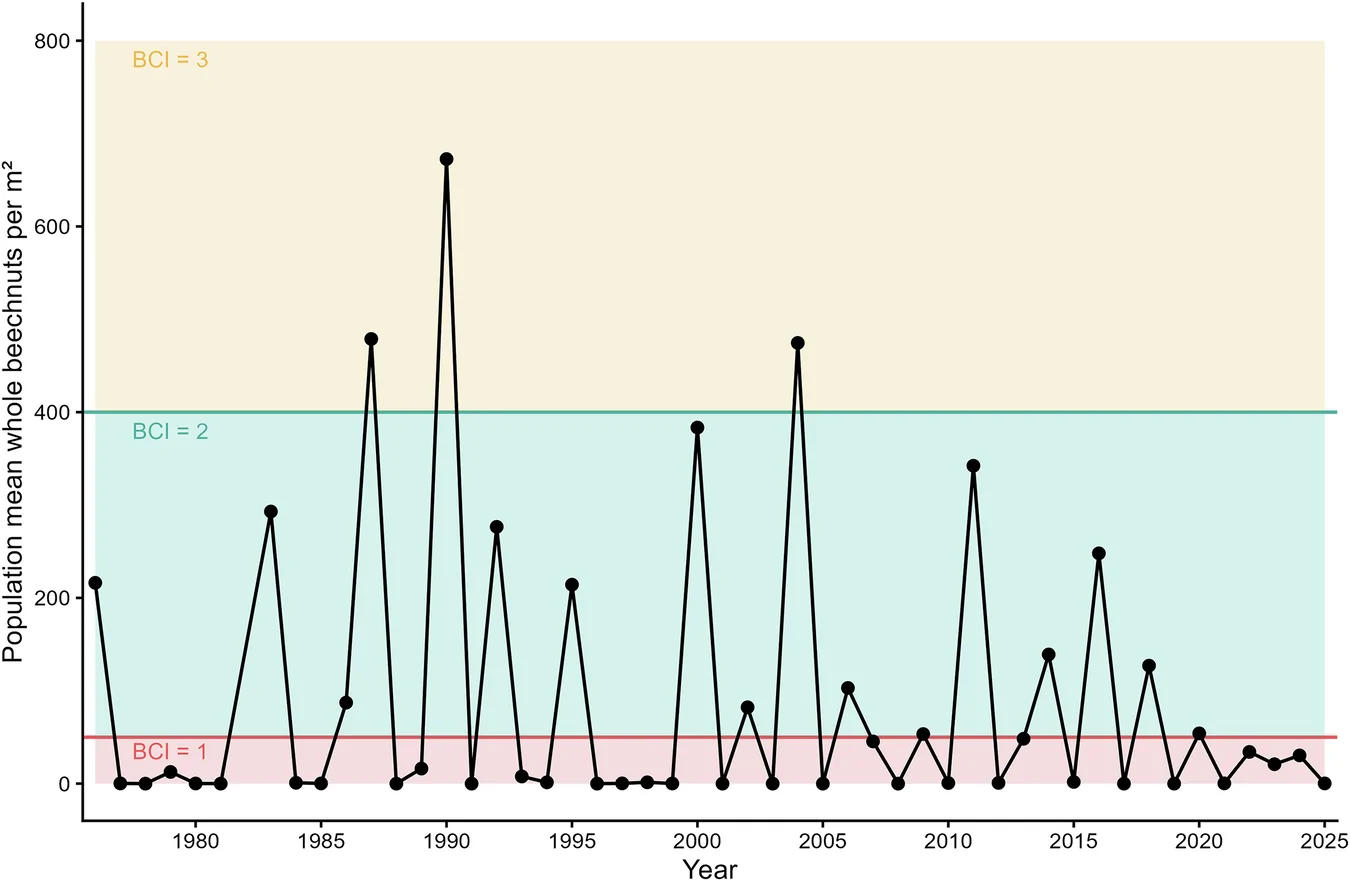
Cut-off point to calculate the beech crop index (BCI) as defined by Perdeck *et al*.^2^ The black dots show the population mean of the number of whole beechnuts per m² per year. The coloured bands indicate which category of the BCI would be assigned to the years. The cut-off points for each level of the index are 50 nuts/m² for BCI = 1, 400 nuts/m² for BCI = 2 and everything above 400 nuts/m² is BCI = 3.

The beech crop index (BCI), that has previously been used in studies on this population, was introduced by Perdeck *et al*.^2^ to make this dataset integrable with an older time series that consists of visual estimates on a nine-point scale by the Dutch State Forestry Service (SBB). Based on the masting dynamics of beeches, with high seeding years commonly followed by low years and some intermediate values, the measured counts of whole nuts per square metre were translated into a three-level index (1 = low, 2 = intermediate and 3 = high). The cut-off points were defined as 0 to 50 nuts/m² for index 1, 50 to 400 nuts/m² for index 2 and >400 nuts/m² for index 3 (Fig. 4). This index was only based on the number of whole nuts, because it was always used in the context of food availability for seed consuming species, for which only filled nuts are important.

If the data are not used together with other seed production data, we recommend to use the original counts instead of calculating the index, as masting patterns are changing with climate change^7,26^, so that the cut-off points might no longer be adequate and less suitable to capture fine-scale variation in beechnut production between years.

### Making data FAIR

Before sharing the data, we enhanced their reusability by making them compliant with the FAIR (findable, accessible, interoperable, reusable) principles^27^, following the workflow described in^28^. This included applying a machine-readable standard to both data and metadata, using ontological terms, where possible, to describe data contents and restructuring the data. For the data, we used Darwin Core^29^, a data standard tailored to biodiversity data, while for the metadata we used the Ecological Metadata language (EML)^30^. We additionally, restructured the data into a Darwin Core Archive^31^, where data contents are organised in a core file and several extension files, which are linked to the core file (see below for more details). Together, these steps allow for easier integration of the dataset with others, easier understandability and less ambiguity, ultimately enhancing the reusability. As we do not use a knowledge representation language, the data is however not fully FAIR, but FAIR enough to be reused in the ecological community (as discussed in more detail in^28^).

## Data Records

The dataset^32^ is available under a CC BY 4.0 licence on DataverseNL, a Dutch installation of Dataverse, and contains a total of 81 individual trees and 12296 plot samples over 50 years. It consists of three tabular data files stored as CSV files using Darwin Core terms as column headers and a separate CSV file that is not in a standard format as a helper file (Include-dates.csv, see below). Following the terminology and structure of a Darwin Core Archive for sampling-event data, the data contents are organised in an event core file (beechcrop_event.csv) and two extension files, occurrence (beechcrop_occurrence.csv) and extended measurement or fact (beechcrop_extendedmeasurementorfact.csv), that are both linked to the core file. The event core stores information about each sampling event and describes a hierarchical event structure. The first and highest event level describes the sampling of an individual tree on a certain date, the second level describes the sampling of one plot of a tree on a certain date and the third and lowest level describes the weighing of individual nuts (or groups of nuts) in the lab. Within the event file, each event is assigned two dates, an eventDate and a verbatimEventDate. While the eventDate describes the calendar day on which the data has been collected, verbatimEventDate contains the year of the season to which this date belongs (if data is collected throughout winter and some collection events are in the following calendar year, they should be assigned to the same year as measurements in October). Additionally, the event core describes details on space, sampling protocol, and sampling effort of each event and contains a unique eventID for each event by which it is linked to the records in both extension files. The occurrence file contains taxonomic and quantitative information of each sampling event and the extended measurement or fact file contains DBH measures and the actual counts and weights of the beechnuts per sampling event. To use this data in analysis, the three tables have to be merged based on their IDs. For more details on the meaning of column names and IDs see Table 1.

**Table 1:** Description of column names in the different files of the Darwin Core Archive.

File name	Column name	Description
beechcrop_event/beechcrop_occurrence	eventID	Unique ID identifying the event to which occurrences and measurements link; central identifier linking all files; describes three hierarchical event levels (see main text)
beechcrop_event	parentEventID	Unique ID identifying the parent event of an event (e.g., measuring a tree on a data is the parent event of measuring the plots of a tree); NA for highest level events
beechcrop_event	verbatimDate	Indicates the season to which a measurement belongs; measurements taken, e.g., in January belong to the season of the previous year
beechcrop_event	samplingProtocol	Specifies the protocol used for the data collection at an event
beechcrop_event	verbatimLocation	Name of the study site
beechcrop_event	fieldNumber	Date-tree-plot specific ID given to entries at data collection; retained to facilitate link to original field books
beechcrop_occurrence/beechcrop_extendedmeasurementorfact	occurrenceID	Unique ID identifying the occurrence of organisms at an event; here it is always one occurrenceID per eventID and can be either one tree for the highest-level event, or a number of nuts for plot level events
beechcrop_occurrence	organismID	Unique ID for each individual tree; NA for occurrences that describe the number of nuts found per plot
beechcrop_occurrence	recordedByID	Unique ID (anonymously) identifying the data collector in the field; IDs are identical to the ones used in *measurementDeterminedBy*; often NA, as identity of observers was rarely recorded
beechcrop_occurrence	occurrenceStatus	Defines presence or absence of an organism (here: always present)
beechcrop_extendedmeasurementorfact	measurementID	Unique ID per measurement; each tree level event has one measurementID, each plot level event nine, and each nut level event up to two
beechcrop_extendedmeasurementorfact	measurementType	Description of the type of measurement using controlled vocabulary where possible
beechcrop_extendedmeasurementorfact	verbatimMeasurementType	Verbatim, simple description of the type of measurement
beechcrop_extendedmeasurementorfact	measurementDeterminedDate	The date on which the measurements were taken
beechcrop_extendedmeasurementorfact	measurementDeterminedBy	Unique ID (anonymously) identifying the data collector in the lab; IDs are identical to the ones used in *recordedByID*; often missing, as identity of observers was rarely recorded
beechcrop_extendedmeasurementorfact	measurementMethod	Describes the method used to obtain the *measurementValue*; specifies the measurement further

File name refers to the names of the CSV files in the dataset in which the column names occur. The description explains the meaning of these columns in this dataset. Note that these column names come with clear definitions in the Darwin Core namespace and are (partly) required by the Darwin Core Archive structure of the data. Columns that clearly indicate their contents (e.g., day, month, decimalLongitude) are not included in the table.

Metadata is stored in two files: an XML file (beechcrop_meta.xml) that contains the structural metadata (i.e., how files are linked) and refers to the URI of each Darwin Core term and a second XML file (beechcrop_EML.xml) using EML to describe the remaining metadata, e.g., data collection, provenance, author and contact details.

Additionally, there is a separate CSV file (Include-dates.csv) that is not part of the collected data but facilitates its usage by indicating which dates to include in analyses of annual beechnut production for years with several sampling dates (see “*Including the correct dates”*). It contains all sampling dates, the number of plots that were sampled at that day and a column indicating which dates to include (i.e., records that have a year in the include column should be included in that year).

While the Darwin Core Archive provides a standardised format that facilitates integration with other datasets, it can be difficult to use in analyses. To facilitate data use, the dataset therefore comes with a short R pipeline (pipeline_DarwinCore-Archive-to-analysis-format.R) that converts the Darwin Core Archive structure into a format more easily usable for data analyses. This pipeline excludes re-sampling plots (SampleTypeID = 3) and those sampling dates that should not be included according to the Include-dates table. If the user wishes to include this data, the script needs to be adapted.

## Technical Validation

Intensive data cleaning was performed before publication of the dataset based on the original field books by manually comparing a random subset of entries in the books and database. When converting the original data into the Darwin Core Archive, data was further checked for the correct number of plots, measurements and occurrences per sampling event, consistency in the data structure, and outliers in measurements were manually checked in the field books. Validation of the counts and weights are not possible, as the original beechnut samples are not available anymore.

## Usage Notes

To use the data as done in previous studies, the beech crop index has to be calculated based on the mean number of whole nuts produced by the population in a year, using the cut-off points provided. As in some, but not in all years, beechnuts were collected on several days, it is important to choose the correct dates for analyses that use annual counts of beechnuts. To facilitate the use of the data, the dataset contains an R pipeline that converts the Darwin Core Archive format of the data into two data frames directly usable for analysis.

## Data Availability

The dataset is available on Dataverse under the https://doi.org/10.34894/TQY74M.

## Code availability

The R script converting the data into a different format more easily usable for analysis is stored along the data on DataverseNL^32^.
